# Prescription Patterns and Adherence to GOLD 2021 Guidelines for Chronic Obstructive Pulmonary Disease Management in Pakistan: A Cross‐Sectional Study

**DOI:** 10.1002/hsr2.73477

**Published:** 2026-11-10

**Authors:** Hafsa Kanwal, Syed Sikandar Shah, Areeg Anwer Ali, Amjad Khan, Umm‐e‐Kalsoom, Saima Mushtaq, Mahwish Rabia, Yu Fang

**Affiliations:** ^1^ Department of Pharmacy Quaid‐i‐Azam University Islamabad Pakistan; ^2^ Department of Clinical Pharmacy and Pharmacology, Rak College of Pharmacy RAK Medical and Health Sciences University Ras Al Khaimah United Arab Emirates; ^3^ Department of Pharmacy The First Affiliated Hospital of Xi'an Jiaotong University Xi'an China; ^4^ Department of Pharmacy Administration and Clinical Pharmacy, School of Pharmacy Health Science Center Xi'an Jiaotong University Xi'an China; ^5^ Department of Pharmaceutical Sciences Pak‐Austria Fachhochshule: Institute of Applied Haripur Pakistan; ^6^ Department of Statistics Government College Women University Sialkot Pakistan

**Keywords:** chronic obstructive pulmonary disease, drug prescriptions, GOLD Guidelines, medication adherence, Pakistan, pulmonologists

## Abstract

**Background and Aims:**

Global Initiative for chronic obstructive lung disease (GOLD) offers evidence‐based recommendations for the management of COPD, but compliance with the guidelines amongst physicians in LMC countries is varied. The objective of this study was to determine the prescribing pattern of inhaled COPD medications in Pakistan and to evaluate the adherence of pulmonologists with the GOLD 2021 guidelines and to assess all the factors that would affect the adherence.

**Methods:**

This cross‐sectional observational study was undertaken at secondary health care facilities Rawalpindi and Islamabad between the period of April–September, 2022. Spirometry, COPD Assessment Test (CAT) and Modified Medical Research Council (mMRC) scores were used to classify outpatients ≥ 40 years old who had COPD confirmed by spirometry into GOLD 2021 ABCD groups. Pharmacological regimens recommended by GOLD were compared with prescribed regimens. Adherence was rated as appropriate, overtreatment or undertreatment. Demographic and clinical variables were correlated with adherence using chi‐square and t‐test.

**Results:**

Among 400 patients (mean age 61.8 ± 10.0 years; 74.8% male), Group B was most frequent (40.3%), followed by C (24.0%), A (23.8%), and D (12.0%). Overall, 57.0% (228/400; 95% CI: 52.1–61.9%) received guideline‐concordant therapy, while 43.0% (172/400; 95% CI: 38.1–47.9%) were inappropriately managed. The 80 patients were considered to be overtreated and 92 patients were considered to be undertreated among the inappropriate prescriptions. Cardiovascular comorbidity was associated with treatment inappropriateness (OR, 1.57; 95% CI, 1.05–2.34; *p* = 0.026), and male sex was also significantly associated with inappropriate prescribing (OR, 2.36; 95% CI, 1.44–3.85; *p* = 0.001).

**Conclusion:**

More than two‐fifths of patients received pharmacological treatment that was not concordant with GOLD 2021 recommendations. Both overtreatment and undertreatment were observed. Strengthening spirometry‐based assessment, clinician education, and access to appropriate inhaled therapies may improve guideline‐concordant COPD management in Pakistan.

## Key Points

- **What is known:** Adherence to guideline‐recommended pharmacological treatment for chronic obstructive pulmonary disease remains inconsistent, particularly in resource‐limited healthcare settings.
- **What is new:** In this cross‐sectional study of 400 patients in Pakistan, 57.0% of prescriptions were concordant with GOLD 2021 recommendations, while 43.0% were inappropriate, comprising both overtreatment and undertreatment.
- **Clinical implications:** Improving spirometry‐based assessment, clinician education, treatment review, and access to appropriate inhaled therapies may support more consistent implementation of GOLD recommendations.

## 1 Background

COPD is a chronic lung disease in which airflow in the lungs is permanently narrowed, the lungs become inflamed and there are frequent worsening of the disease (exacerbations) that cause permanent damage to the structure of the lungs. It is a leading cause of chronic morbidity and mortality globally, and has a wide spectrum of health, social and economic implications [1]. According to the World Health Organization (WHO), COPD is one of the three main causes of death in the world, responsible for the deaths of an estimated 3,230,000 people every year. The Global Burden of Disease (GBD) 2019 study found that more than 80% of these deaths take place in low‐and middle‐income countries (LMICs) where inadequate health systems, increased exposure to risk factors and delayed diagnosis exacerbates the burden [2, 3]. COPD is a polyetiological disease. In high‐income countries, tobacco smoking is the major risk factor, while in LMICs, other exposures, including indoor biomass fuel combustion, occupational dust and chemicals, and ambient air pollution are also important [4].

COPD is increasingly becoming a common problem in South Asia, due to the escalating rate of urbanization and industrialization, which has led to worsening air quality. COPD can also cause significant functional impairment, causing individuals to be less able to work and to have a reduced quality of life. It generates economic costs through direct costs (including frequent hospitalisations, long term drug consumption, and diagnostic tests) and indirect costs (including loss of productivity, disability and premature death) [5, 6]. For Pakistan whose population is large and growing, COPD is a big problem. In 2019, an estimate placed the prevalence in adults at 2.1%, or millions of affected adults. The prevalence may be underestimated, however, because of underdiagnosis, limited access to diagnostic testing (e.g. spirometry) and poor public knowledge of the disease. High smoking prevalence, heavy reliance on biomass fuel for cooking and home heating in rural areas, at work exposures, and ambient air pollution in urban areas [7] all add to the country's risk profile.

Socio‐economic factors, poor training on chronic disease management at primary and secondary levels and a shortage of critical respiratory medicines further complicate healthcare delivery [8]. In 1998, the Global Initiative for Chronic Obstructive Lung Disease (GOLD) was created by a partnership between the National Heart, Lung, and Blood Institute (NHLBI) and WHO. It is tasked with creating recommendations and guidelines arising from evidence to enhance the prevention, diagnosis and management of COPD [9, 10, 11]. Since its first major report in 2001, GOLD has produced strategy documents every year, which are regarded as the international standard for COPD care. GOLD originally used spirometric findings (post bronchodilator) alone to classify COPD severity as Grades 1–4, and not patient‐reported symptom burden or exacerbation history to guide treatment decisions (Patel et al., 2019). But studies in the past 20 years have shown that there was not always a strong association between airflow limitation severity and symptoms or the exacerbation risk [12, 13, 14, 15, 16, 17, 18, 19].

This knowledge resulted in a landmark shift in 2011 when the ABCD assessment tool was introduced, a tool which combines symptom burden (based on the Modified Medical Research Council [mMRC] dyspnea scale or COPD Assessment Test [CAT]) with exacerbation history to inform treatment choice [19]. In 2017, this was further refined with the removal of spirometric grading from the treatment algorithm and a focus on the importance of individualising pharmacotherapy based on clinical presentation [20]. The 2021 GOLD update reaffirmed the guideline to consider using dual long‐acting bronchodilators (LABA + LAMA) in patients with high symptom burden, to consider the use of inhaled corticosteroids (ICS) for patients with frequent exacerbations and elevated eosinophil‐levels and strongly discouraged the use of inhaled corticosteroid monotherapy [21].

GOLD recommendations have been widely disseminated, but there is little evidence of their uptake in health care systems. International studies have revealed the adherence rate varies from low of 29.7% in Tunisia to high of 79% in Romania, with many countries reporting adherence of around 50% to 60% [22, 23]. Another recurring issue is undertreatment in high‐risk patients (C and D), with no treatment or wrong treatment type being used, such as ICS or triple therapy, which increases the risk of patients developing pneumonia [24]. On the other hand, undertreated high‐risk patients (C and D) are at risk of recurrent exacerbations, hospitalizations, and rapid loss of lung function [25]. Physician‐related factors refer to the lack of knowledge about the updated guidelines, physician's personal clinical experiences, misconceptions regarding the effectiveness of specific classes of drugs, and lack of use of standardized assessment tools such as CAT and mMRC [26]. Regardless of the obstacles encountered, inadequate adherence has a detrimental impact on patients' clinical outcomes, heightening the risk of unforeseen acute exacerbations of COPD, and positive treatment outcomes [22, 27, 28]. Increased adherence, in contrast, is clinically and functionally beneficial, as well as economically beneficial, that decreases direct and indirect health care costs [15, 26, 29, 30, 31]. The majority of researches on physician adherence to the GOLD guidelines have been undertaken at multiple levels—regional, and international [1, 10, 12, 15, 17, 19, 28, 29, 32]., still there is no study to assess the adherence to GOLD guidelines has been conducted in Pakistan. The study results are contradictory and the most variable of all studies [8, 33] making it impossible to extrapolate the results of the study to Pakistan. These studies have been carried out not only amongst pulmonologists, but also amongst specialists in treating COPD [34] and assessing various COPD‐GOLD guidelines [19]. In a study, recommendations were shown to be relevant and the need to streamline their dissemination for their implementation in healthcare settings was highlighted [15]. Greater interaction between health care providers and policy‐makers involved in the implementation of guidelines is critical. This partnership will help to identify factors that interfere with adherence and make recommendations that address the needs of routine healthcare delivery [9]. In light of these findings, it was important to evaluate the level of awareness and use of COPD management guidelines among the pulmonologists of Pakistan. This is especially relevant because a 2019 study estimated that the prevalence of this condition is 2.1% amongst adults in the country generally [35]. This study has focused only on the practices of physicians with regard to COPD management.

## 2 Methods

### 2.1 Study Design, Setting

A cross‐sectional observational study was conducted from April 1 to September 30, 2022, in 3 secondary‐level healthcare facilities in Rawalpindi and Islamabad, Pakistan. The participating facilities included two public‐sector and one private‐sector healthcare facility providing outpatient respiratory care. Patients were assessed by board‐certified pulmonologists. The study was reported in accordance with the Strengthening the Reporting of Observational Studies in Epidemiology (STROBE) guidelines.

### 2.2 Study Population and Eligibility Criteria

Patients aged ≥ 40 years with confirmed COPD who attended the participating outpatient facilities during the study period were eligible. COPD was confirmed by spirometry according to the study eligibility criteria. Patients were included if they were clinically stable and had no acute COPD exacerbation during the preceding 4 weeks. Patients were excluded if they were hospitalized, had incomplete clinical or prescription records, had asthma or asthma‐COPD overlap, or were experiencing an acute exacerbation at the time of assessment. Asthma‐COPD overlap was excluded on the basis of a documented history of asthma, significant bronchodilator reversibility, or a physician diagnosis of asthma‐COPD overlap.

### 2.3 Sample Size Calculation and Sampling Method

The required sample size was calculated using an anticipated proportion of 50%, a 5% margin of error, and a 95% confidence level, resulting in a minimum sample of 384 participants. During the study period, 504 potentially eligible patients were screened. After application of the eligibility criteria, 400 patients were included in the final analysis. Consecutive sampling was used to recruit eligible participants. The sample size was sufficient for estimating the overall proportion of guideline‐concordant prescribing but was not specifically powered to detect differences within individual subgroups.

### 2.4 Patient Consent Statement

All of the individual participants in the study signed informed consent agreements. The participants were guaranteed confidentiality and their participation was made voluntary, and the data were anonymised before analysis.

### 2.5 Data Collection Procedures

Information was gathered in a prospective manner with a case report form (CRF) created specifically for this study to standardise the data collection process. The information was gathered from patient's medical records by the research team that comprised trained Clinical Pharmacists and Research Assistants with the aim of filling the missing information by direct interaction with patients. Demographic information included was age, gender and place of residence (urban or rural). Smoking history was defined as current smoking, former smoking, or never smoking. Clinical variables included spirometric GOLD grade [1, 2, 3, 4], symptom severity using the COPD Assessment Test (CAT) and the Modified Medical Research Council (mMRC) dyspnea scale, and exacerbation history within the preceding year. The presence of cardiovascular comorbidities was defined as a documented diagnosis of ischemic heart disease, heart failure, arrhythmia, or other chronic cardiac conditions.

### 2.6 GOLD Classification and Operational Definitions

Patients were classified according to the GOLD 2021 ABCD framework using CAT, mMRC, and exacerbation history. A CAT score ≥ 10 and/or mMRC grade ≥ 2 indicated higher symptom burden, whereas CAT < 10 and mMRC 0–1 indicated lower symptom burden. Exacerbation risk was considered low with 0–1 exacerbation without hospitalization and high with ≥ 2 exacerbations or ≥ 1 hospitalization in the previous year. These measures were then combined to assign patients to GOLD Groups A–D. The appropriateness of pharmacological treatment was assessed by comparing prescribed inhaled regimens with GOLD 2021 recommendations for each group. The following operational definitions were applied: Appropriate therapy: Prescribed regimen matches the first‐line or alternative option recommended by GOLD 2021 for the patient's group. Overtreatment: Prescribed regimen exceeds the recommended intensity (e.g., triple therapy in Group A). Undertreatment: The prescribed regimen was below the treatment intensity recommended by GOLD 2021 for the corresponding patient group Table 1. Cardiovascular comorbidity: Presence of at least one documented cardiac condition in the medical record.

**Table 1:** Treatment algorithm according to the GOLD guidelines.

	Appropriateness		Inappropriateness	
Group	First choice	Alternative choice	Under‐treatment	Over‐treatment
A	A bronchodilator	Change the bronchodilator	No bronchodilator prescribed	LABA + LAMA, ICS or any combination treatment
B	LAMA or LABA	LAMA + LABA	Only short‐acting bronchodilator	LAMA + LABA + ICS, ICS + LABA, ICS + LAMA
C	LAMA	LAMA + LABA or ICS + LABA	Only ICS or LABA or SABA	ICS + LAMA, ICS + LABA + LAMA
D	LAMA + LABA or LAMA or ICS + LABA	If still exacerbation present ICS + LABA + LAMA	Only ICS or SABA or LABA, ICS + LAMA	

*Note:* SABA – Short‐acting β_2_‐agonist, LABA – Long‐acting β_2_‐agonist, SAMA – Short‐acting muscarinic antagonist, LAMA – Long‐acting muscarinic antagonist, ICS – Inhaled corticosteroid.

### 2.7 Assessment of Adherence and Quality Control

To ensure consistency in data extraction and classification, two investigators independently reviewed the clinical and prescription data. Disagreements were resolved through discussion and consensus. Inter‐rater agreement for treatment classification was assessed using Cohen's kappa in a random sample of 50 patients, with a kappa value of 0.89 indicating high agreement.

## 3 Statistical Analysis

Data entry was done on MS Excel software and statistical analysis was done using IBM SPSS Statistics 25.0 (IBM Corp., Armonk, NY, USA). Normality of continuous variables were assessed using Shapiro–Wilk test. Means and standard deviations (SDs) are used to summarize continuous variables that are distributed normally (e.g., BMI, weight, age). If continuous data is not normally distributed, then median and interquartile range (IQR) would be used to summarize it. Categorical variables shown as frequencies and percentages. Association between categorical variables was investigated using a Chi square test. Odds ratios (ORs) with 95% confidence intervals (CIs) were calculated to quantify univariate associations. No formal interaction terms between predictor variables were evaluated. The assessment of overall adherence to GOLD recommendations constituted the primary prespecified analysis, whereas analyses examining associations between adherence and demographic or clinical characteristics were considered exploratory. All statistical tests were two‐sided, and a *p*‐value < 0.05 was considered statistically significant.

## 4 Results

During the study period, 504 potentially eligible patients were screened. After application of the eligibility criteria, 400 patients were included in the final analysis. The excluded patients comprised those with acute exacerbation, incomplete clinical or prescription information, or asthma/asthma‐COPD overlap.

**Baseline characteristics** The study included 400 patients with a mean age of 61.8 ± 10.0 years, of whom 74.8% were male. Most participants were from urban areas (62.0%). Current smokers accounted for 46.3% of the study population, followed by never smokers (32.5%) and former smokers (21.3%). Based on spirometric GOLD grade, 41.8% of patients were classified as Grade 2, 33.0% as Grade 1, 17.3% as Grade 3, and 8.0% as Grade 4. According to the GOLD 2021 ABCD classification, Group B was the most frequent (40.3%), followed by Group C (24.0%), Group A (23.8%), and Group D (12.0%). Cardiovascular comorbidities were reported in 46.5% of patients, while 37.5% had other comorbidities and 13.5% had no reported comorbidity. Baseline characteristics are presented in Table 2.

**Table 2:** Demographic and clinical characteristics of COPD patients (*n* = 400).

Characteristic	*n*	% Of total (*n* = 400)	% Within category
Age (years), Mean ± SD	61.82 ± 10.02	—	—
Gender			
Male	299	74.8	—
Female	101	25.2	—
Residence			
Urban	248	62.0	—
Rural	152	38.0	—
Smoking history			
Current smoker	185	46.3	—
Former smoker	85	21.3	—
Never smoker	130	32.5	—
Spirometric GOLD grade			
Grade 1	132	33.0	—
Grade 2	167	41.8	—
Grade 3	69	17.3	—
Grade 4	32	8.0	—
GOLD 2021 group			
Group A	95	23.8	—
Group B	161	40.3	—
Group C	96	24.0	—
Group D	48	12.0	—
Comorbidities			
Cardiovascular comorbidity (total)	**186**	**46.5**	**100.0**
Ischemic heart disease (IHD)	72	18.0	38.7
Heart failure (HF)	40	10.0	21.5
Cardiac arrhythmia	32	8.0	17.2
Cerebrovascular disease	22	5.5	11.8
Peripheral arterial disease	20	5.0	10.8
Other comorbidities (total)	**150**	**37.5**	**100.0**
Hypertension*	48	12.0	32.0
Diabetes mellitus	42	10.5	28.0
Chronic kidney disease (CKD)	20	5.0	13.3
Dyslipidemia	18	4.5	12.0
Osteoporosis	12	3.0	8.0
Anxiety/depression	10	2.5	6.7
No recorded comorbidity	54	13.5	—

*Note:* GOLD 2021 group classification was based on symptom burden assessed using CAT and/or mMRC together with the documented history of COPD exacerbations during the preceding 12 months. CAT ≥ 10 and/or mMRC ≥ 2 indicated higher symptom burden, while CAT < 10 and mMRC 0–1 indicated lower symptom burden. Hypertension is listed under “Other comorbidities” when no documented cardiovascular disease is present. If hypertension coexists with IHD or HF, it is classified under cardiovascular comorbidity. Percentages may not total 100% due to rounding and because patients may have multiple comorbidities.

Abbreviations: CKD, chronic kidney disease; HF, heart failure; IHD, ischemic heart disease.

### 4.1 Medication Prescribing Patterns

The distribution of prescribed inhaled pharmacological regimens across GOLD 2021 groups is presented in Table 3. LABA‐containing regimens were the most frequently prescribed overall (72.5%). In Group A, LABA monotherapy (40.0%) and LAMA monotherapy (18.9%) were commonly prescribed. In Group B, LABA monotherapy (35.4%) and LAMA + LABA dual therapy (29.8%) were the most frequently observed regimens. Groups C and D showed greater use of dual and triple inhaled therapy. Triple therapy (LAMA + LABA + ICS) was prescribed in 19.8% of Group C patients and 43.8% of Group D patients. ICS monotherapy was uncommon and was prescribed in 1.9% of Group B patients.

**Table 3:** Distribution of prescribed COPD pharmacological regimens by GOLD group (*n* = 400).

Medication regimen	Group A *n* (%)	Group B *n* (%)	Group C *n* (%)	Group D *n* (%)	Total *n* (%)
Monotherapy					
LABA	38 (40.0)	57 (35.4)	16 (16.7)	5 (10.4)	116 (29.0)
LAMA	18 (18.9)	20 (12.4)	12 (12.5)	4 (8.3)	54 (13.5)
Dual therapy					
LABA + LAMA	10 (10.5)	48 (29.8)	24 (25.0)	12 (25.0)	94 (23.5)
LABA + ICS	12 (12.6)	14 (8.7)	10 (10.4)	6 (12.5)	42 (10.5)
Triple therapy					
LAMA + LABA + ICS	7 (7.4)	15 (9.3)	19 (19.8)	21 (43.8)	62 (15.5)
Short‐acting regimens					
SABA	5 (5.3)	4 (2.5)	3 (3.1)	0 (0.0)	12 (3.0)
SAMA	3 (3.2)	2 (1.2)	2 (2.1)	0 (0.0)	7 (1.8)

*Note:* LABA – long‐acting β_2_‐agonist; LAMA – long‐acting muscarinic antagonist; ICS – inhaled corticosteroid; SABA – short‐acting β_2_‐agonist; SAMA – short‐acting muscarinic antagonist.

### 4.2 Adherence to GOLD Guidelines

Comparison of prescribed inhaled regimens with the GOLD 2021 recommendations showed that 228 of 400 patients (57.0%; 95% CI: 52.1–61.9%) received guideline‐concordant treatment, whereas 172 patients (43.0%; 95% CI: 38.1–47.9%) received treatment classified as inappropriate. Among the 400 patients, 80 (20.0%; 95% CI: 16.1–23.9%) were classified as receiving overtreatment and 92 (23.0%; 95% CI: 18.9–27.1%) as receiving undertreatment. Undertreatment was more frequently observed in Groups C and D, whereas overtreatment was more frequently observed in Groups A and B. The distribution of treatment appropriateness across GOLD groups is presented in Table 4.

**Table 4:** Frequency of under‐treatment and over‐treatment by GOLD 2021 group (*n* = 400).

Prescribing category	*n*	%	95% CI (%)
Guideline‐concordant therapy	228	57.0	52.1–61.9
Inappropriate therapy	172	43.0	38.1–47.9
Overtreatment^a^	80	20.0	16.1–23.9
Undertreatment^a^	92	23.0	18.9–27.1

a Overtreatment and undertreatment percentages are expressed as proportions of the total study population (*n* = 400). Confidence intervals were calculated at the 95% level using the binomial distribution.

### 4.3 Factors Associated with Adherence

Associations between patient characteristics and adherence status are shown in Table 5. Cardiovascular comorbidity was associated with non‐adherence OR 1.57 (95% CI 1.05–2.34). Male sex was also associated with treatment appropriateness (OR 2.36, 95% CI: 1.44–3.85; χ^2^ = 12.074, *p* = 0.001). No statistically significant associations were observed between treatment appropriateness and age (*p* = 0.18), residential location (*p* = 0.260), or GOLD group (*p* = 0.50). Among cardiovascular comorbidities, heart failure and arrhythmia were associated with higher odds of inappropriate treatment. All reported odds ratios were unadjusted estimates derived from univariate analyses, and no formal statistical interactions between predictor variables were assessed.

**Table 5:** Univariate associations between patient characteristics and inappropriate COPD therapy.

Predictor	Level	Appropriate *n* (%)	Inappropriate *n* (%)	OR	95% CI	*p*‐value
Age (years)	Mean ± SD	61.25 ± 10.11	62.60 ± 9.88	—	—	0.183
Gender	Male	157 (39.2)	142 (35.5)	2.36	1.44–3.85	0.001*
	Female	73 (18.2)	28 (7.0)	Ref	—	—
Residence	Urban	148 (37.0)	100 (25.0)	0.79	0.53–1.19	0.260
	Rural	82 (20.5)	70 (17.5)	Ref	—	—
Cardiovascular comorbidity (overall)	Yes	96 (24.0)	90 (22.5)	1.57	1.05–2.34	0.026*
Ischemic heart disease	Yes	38 (52.8)	34 (47.2)	1.30	0.78–2.16	0.321
Heart failure	Yes	16 (40.0)	24 (60.0)	2.25	1.16–4.38	0.015*
Arrhythmia	Yes	12 (37.5)	20 (62.5)	2.48	1.18–5.22	0.014*
Cerebrovascular disease	Yes	9 (40.9)	13 (59.1)	2.08	0.87–4.98	0.095
Peripheral arterial disease	Yes	10 (50.0)	10 (50.0)	1.41	0.55–3.46	0.457
GOLD risk (ABCD)	High (C/D)	86 (21.5)	58 (14.5)	0.87	0.57–1.31	0.500
	Low (A/B)	144 (36.0)	112 (28.0)	Ref	—	—

*Note:* ORs represent unadjusted estimates from univariate logistic regression analyses. No interaction terms between predictor variables were included in the model. Because some cardiovascular comorbidity subgroups contained relatively small numbers of participants, the corresponding OR estimates may be imprecise and should be interpreted cautiously. Sparse‐data effects may result in wide confidence intervals and unstable estimates. Odds ratios (ORs) with 95% confidence intervals (CIs) were calculated to quantify univariate associations. No formal interaction terms between predictor variables were evaluated.

## 5 Discussion

This study aimed to assess the compliance with the GOLD (2021) pharmacological treatment guidelines for COPD in secondary level healthcare facilities of Pakistan with focus on identifying factors associated with inappropriate prescription and evaluate the effect of cardiovascular comorbidities. The overall adherence rate was 57.0% (95% CI: 52.1–61.9%), meaning that almost half of the COPD patients were not well treated according to the GOLD recommendations, and under‐ and overtreatment were common. Our results are consistent with recent studies conducted after the GOLD 2021 update, which have reported suboptimal adherence to guideline‐recommended COPD therapy, with adherence rates generally ranging from 57% to 64% in South Korea [12], Switzerland [36] and Hong Kong [12] respectively. A major concern among many healthcare settings is overtreatment in patients with low‐risk and inappropriate use of inhaled corticosteroids. These results indicate that the implementation of the new GOLD recommendations in clinical practice is still difficult, especially in low‐resources areas [37, 38]. As seen in previous studies, more patients in GOLD A and B were over‐treated and more patients in GOLD C and D were under‐treated [39, 40]. This may be caused by a variety of factors. Clinicians commonly start triple therapy in patients who have symptoms and have little risk for exacerbation, but feel that the more aggressive therapy might benefit symptoms, although there are no guidelines for this [41]. On the other hand, undertreatment in higher‐risk groups can be related to medication expenses, scarcity of long‐acting bronchodilators in public‐sector formularies or clinical risk aversion due to coexisting medical conditions [42]. Furthermore, prescription knowledge of older prescribing habits, inadequate awareness about updates to GOLD every year and a lack of structured tools to assess symptoms and exacerbations may lead to therapeutic misclassification [36]. These prescribing practices have clinical implications that need to be understood. Overtreatment is especially likely when inhaled corticosteroid (ICS)‐based treatment is used unnecessarily in people with low‐risk conditions (Groups A and B), which can lead to unwanted side effects such as oral candidiasis and risk of pneumonia and to a patient's higher costs of treatment without a significant clinical benefit. On the other hand, undertreatment (Groups C and D) could result in suboptimal symptom control, higher exacerbation rates, faster rates of decline in lung function, worse quality of life and higher hospitalization risks. These results highlight the need to match the intensity of treatment with recommendations from guidelines to maximize the outcomes for the patient.

What's more, there is a subgroup analysis of cardiovascular comorbidities. Inappropriate prescribing was significantly associated with heart failure and cardiac arrhythmia, but there was a non‐significant association with ischemic heart disease, cerebrovascular disease and peripheral arterial disease. Deliberate avoidance of some β_2_‐agonists may be a cause for their use in heart failure and arrhythmia patients being under treated, because these drugs are known to have a potential for adverse effects like tachycardia or arrhythmia [43, 44]. Otherwise, these patients may be on higher doses of the inhaled corticosteroid‐containing medications because of the perceived risk of exacerbations, even if there is no history of exacerbation [45]. In contrast, neither ischemic heart disease nor cerebrovascular disease had as direct an influence on choice of inhaler, and so had weaker associations. The results are consistent with previous studies that demonstrated that adherence to COPD guidelines is complex when cardiovascular disease is present, and that the prescribing should be tailored to these patients [46, 47]. On the pathophysiological level, systemic inflammation, oxidative stress, and endothelial dysfunction are shared mechanisms that could affect disease progression and therapeutic responsiveness in COPD, and may be responsible for the link with CVD [48]. This interaction of the biological processes provides a basis for integrated care models that treat COPD and CVD together versus separately [44, 46].

The results suggest that there is an immediate need for targeted interventions from a healthcare systems perspective. There is recent evidence that prescription audit (as part of routine care) and CDES integration are associated with better GOLD adherence and facilitating the implementation of new treatments [37]. Inhaler safety in cardiac patients should be addressed through educational programs that highlight guideline compliance in the presence of comorbidities, and misconceptions about inhaler safety [49]. Tele‐clinical support by incorporating electronic tools that aid clinicians in real‐time decisions about aligning the prescription with GOLD criteria could be beneficial [41]. Combined management by both pulmonologists and cardiologists may be the best approach for treating those with both COPD and cardiovascular disease [44]. Moreover, increasing access and affordability of LABD in the public sector may decrease under‐treatment among higher risk populations [43]. Access to health services and costs factors in Pakistan might also influence prescribing patterns. Long‐acting bronchodilators and combination inhalers are important barriers in low‐resource settings that must be addressed. LABA/LAMA and triple therapy inhalers will be less readily available in public health services in Pakistan and could be a heavy cost to patients who do not have comprehensive insurance coverage. These socioeconomic barriers could be responsible for nonadherence to guidelines and should be further studied. The availability of health policy approaches to cost reduction, access to medicine, and increased insurance coverage can have a significant impact on adherence rates. Overall, our results indicate a high priority of ongoing physician training, the use of assessment tools like spirometry and CAT, the improvement of comorbidity screening and locally tailored approaches to improve adherence to the GOLD guideline. The use of essential inhalers should be supported by the policy and the GOLD updates should be disseminated throughout Pakistan to optimize COPD care. Enhancing interdisciplinary communication between pulmonologists, primary care physicians, and pharmacists might further support good documentation and continuity of care as well as evidence‐based prescribing.

This study was conducted using the GOLD 2021 ABCD classification, but the results will be applicable to the new ABE classification in GOLD 2023. Patterns of over‐ and under‐treatment observed suggests that the assessment of symptoms and exacerbations remain crucial in determining treatment decisions, as they are in the ABE approach. Our findings indicate that ensuring proper adherence to guideline‐based treatment and decrease in inappropriate prescriptions should continue to be priorities when moving from ABCD to ABE classification.

## 6 Conclusion

The findings of this study show suboptimal adherence with the 2021 GOLD guidelines in managing COPD in Pakistan (57%), with significant over and under treatment of those at low risk and high risk, respectively. Cardiovascular comorbidity and gender were significantly associated with treatment appropriateness. The prescribing patterns observed suggest a need to enhance the provision of structured assessment of GOLD and guideline‐concordant treatment in the real world. These results highlight the need for more robust clinical education programs, the consistent application of assessment tools, policy changes to increase accessibility to inhalers and adherence to the changing recommendations of the GOLD—in particular the ABE classification – to provide evidence‐based, equitable COPD care.

## 7 Limitations

There are a number of limitations of this study. First, because prescriptions were excluded from those written by primary care physicians and junior doctors, this may limit the applicability of the results to non‐specialist settings. The observed rate of non‐adherence may be higher, as pulmonologists are more likely to be familiar with GOLD guidelines and have access to and capability with diagnostic tools such as spirometry. Secondly, medication cost and health insurance coverage were not recorded as these might influence prescribing practices in a low and middle income country such as Pakistan. Third, although patients with Asthma‐COPD Overlap (ACO) were not included, borderline cases or undiagnosed patients may have been included in the sample. We were also unable to assess prior treatment history and response to treatment which may have resulted in over treatment in stable patients who were treated with escalated therapy in the prior treatment. Furthermore, only univariate analyses were performed and multivariable adjustment for potential confounders was not performed, so that the reported odds ratios are considered as unadjusted associations and residual confounding cannot be excluded. Finally, the sample size was large enough to provide estimates of adherence with the guidelines, but was not specifically designed for analyses of subgroups, including GOLD Group D and specific cardiovascular comorbidities, and so may have not had sufficient power and may have been vulnerable to type II errors.

No further exclusion or sensitivity analyses were carried out for individual comorbidities. Patients with cardiovascular diseases or other chronic conditions were retained in the study as these are common in COPD and relevant to the concept of treatment appropriateness, but the absence of sensitivity analyses for each of these conditions precludes a robust assessment of the robustness of these associations. Therefore, the results of the sub groups should be considered exploratory. Prescribing evaluation per GOLD 2021 was compared but not in relation to the GOLD 2023 ABE framework, however, symptom burden and exacerbation risk continue to be important factors influencing treatment decisions, leading to overtreatment and undertreatment. Patients were not sampled but recruited consecutively from 3 secondary level healthcare facilities, which reduces the generalizability of the findings to the larger population of COPD patients in Pakistan and the cross sectional design does not allow for causal inferences. A few Table 5 subgroups had small cell numbers that might have resulted in sparse‐data effects, unstable or inflated odds ratios, and imprecise confidence intervals. Thus, caution must be taken with these results and there is a need for future studies with sufficient power to use penalized‐likelihood methods [50, 51].

## Author Contributions

**Hafsa Kanwal:** conceptualization, methodology, writing – original draft. **Syed Sikandar Shah:** methodology. **Areeg Anwer Ali:** data curation, investigation. **Amjad Khan:** conceptualization, supervision, methodology, resources. **Saima Mushtaq:** writing – original draft, project administration. **Mahwish Rabia:** formal analysis. **Yu Fang:** supervision.

## Ethics Statement

The study protocol was approved by the Bio‐Ethical Committee of Quaid‐i‐Azam University, Islamabad, Pakistan (Ref No. BEC‐FBS‐QAU2021‐269), and from Holy Family Hospital, Rawalpindi, Pakistan (Ref No. 63/IREF/RMU/2021). All procedures involving human participants were conducted in accordance with the ethical standards of the institutional and national research committees.

## Conflicts of Interest

The authors declare no conflicts of interest.

## Transparency Statement

Dr. Amjad Khan affirms that this manuscript is an honest, accurate, and transparent account of the study being reported; that no important aspects of the study have been omitted; and that any discrepancies from the study as planned have been explained.

## Data Availability

The datasets generated and/or analyzed during the current study are not publicly available due to institutional policy and patient confidentiality but are available from the corresponding author upon reasonable request.

## References

[1] C. F. Vogelmeier , G. J. Criner , F. J. Martinez , et al., “Global Strategy for the Diagnosis, Management, and Prevention of Chronic Obstructive Lung Disease 2017 Report. GOLD Executive Summary,” American Journal of Respiratory and Critical Care Medicine 195, no. 5 (2017): 557–582, 10.1164/rccm.201701-0218PP. 28128970

[2] H. A. H. Albitar and V. N. Iyer , “Adherence to Global Initiative for Chronic Obstructive Lung Disease Guidelines in the Real World: Current Understanding, Barriers, and Solutions,” Current Opinion in Pulmonary Medicine 26, no. 2 (2020): 149–154, 10.1097/MCP.0000000000000655. 31834001

[3] H. Kanwal , S. Khan , G. E. Eldesoky , S. Mushtaq , and A. Khan , “Management of COPD and Comorbidities in COPD Patients by Dispensing Pharmaceutical Care Following Global Initiative for Chronic Obstructive Lung Disease‐Guidelines (GOLD Guidelines 2020): A Study Protocol for a Prospective Randomized Clinical Trial,” Heliyon 9, no. 11 (2023): e21539, 10.1016/j.heliyon.2023.e21539. PMC10628705 37942165

[4] S. Kotlyarov , “Analysis of the Comorbid Course of Chronic Obstructive Pulmonary Disease,” Journal of Personalized Medicine 13, no. 7 (2023): 1179, 10.3390/jpm13071179. PMC10381164 37511792

[5] A. Alshabanat , “Impact of a COPD Comprehensive Case Management Program on Hospital Length of Stay and Readmission Rates,” International Journal of Chronic Obstructive Pulmonary Disease 12 (2022): 961–971. 10.2147/COPD.S124385 PMC5367737 28356728

[6] D. Donesky , Oxford Textbook of Palliative Nursing (2019), 217–229. Dyspnea, Cough, and Terminal Secretions.

[7] S. M. Martins , A. P. Dickens , W. Salibe‐Filho , et al., “Accuracy and Economic Evaluation of Screening Tests for Undiagnosed COPD among Hypertensive Individuals in Brazil,” NPJ Primary Care Respiratory Medicine 32, no. 1 (2022): 55, 10.1038/s41533-022-00303-w. PMC9747958 36513683

[8] J. Sehl , J. O'doherty , R. O'connor , B. O'sullivan , and A. O'regan , “Adherence to COPD Management Guidelines in General Practice? A Review of the Literature,” Irish Journal of Medical Science (1971−) 187, no. 2 (2018): 403–407, 10.1007/s11845-017-1651-7. 28735500

[9] D. L. Ardelean , L. Iulia , R. Popescu , et al., “Evaluation of COPD Patients Using CAT‐COPD assessment test.” Pneumologia (Bucharest, Romania: (2012). 61, 221–229. 4. 23424946

[10] C. Cojocaru , M. I. Marian , and E. Cojocaru , “La Perception de la Fatigue Chez les Patients avec Broncho‐Pneumonie Chronique Obstructive,” Revue des Maladies Respiratoires 29 (2012): A58, 10.1016/j.rmr.2011.10.972.

[11] V. X. Han , S. Patel , H. F. Jones , et al., “Maternal Acute and Chronic Inflammation in Pregnancy Is Associated With Common Neurodevelopmental Disorders: A Systematic Review,” Translational Psychiatry 11, no. 1 (2021): 71, 10.1038/s41398-021-01198-w. PMC7820474 33479207

[12] T.‐O. Kim , H.‐J. Shin , Y.‐I. Kim , et al., “Adherence to the GOLD Guideline in COPD Management of South Korea: Findings From KOCOSS Study 2011–2018,” Chonnam Medical Journal 55, no. 1 (2019): 47–53, 10.4068/cmj.2019.55.1.47. PMC6351324 30740340

[13] R. Sharif , C. R. Cuevas , Y. Wang , M. Arora , and G. Sharma , “Guideline Adherence in Management of Stable Chronic Obstructive Pulmonary Disease,” Respiratory Medicine 107, no. 7 (2013): 1046–1052, 10.1016/j.rmed.2013.04.001. 23639271

[14] A. Corrado and A. Rossi , “How Far Is Real Life From COPD Therapy Guidelines? An Italian Observational Study,” Respiratory Medicine 106, no. 7 (2012): 989–997, 10.1016/j.rmed.2012.03.008. 22483189

[15] E. Sen , S. Z. Guclu , I. Kibar , et al., “Adherence to GOLD Guideline Treatment Recommendations Among Pulmonologists in Turkey,” International Journal of Chronic Obstructive Pulmonary Disease 10 (2015): 2657–2663. 10.2147/COPD.S85324 PMC4686224 26715844

[16] O. Turan , J. C. Emre , S. Deniz , A. Baysak , P. A. Turan , and A. Mirici , “Adherence to Current COPD Guidelines in Turkey,” Expert Opinion on Pharmacotherapy 17, no. 2 (2016): 153–158, 10.1517/14656566.2016.1115482. 26629809

[17] M.‐J. Hsieh , S.‐Y. Huang , T.‐M. Yang , et al., “The Impact of 2011 and 2017 Global Initiative for Chronic Obstructive Pulmonary Disease (GOLD) Guidelines on Allocation and Pharmacological Management of Patients With COPD in Taiwan: Taiwan Obstructive Lung Disease (TOLD) Study,” International Journal of Chronic Obstructive Pulmonary Disease 13 (2018): 2949–2959, 10.2147/COPD.S176065. PMC6165725 30310271

[18] B. Ding , M. Small , and U. Holmgren , “A Cross‐Sectional Survey of Current Treatment and Symptom Burden of Patients With Copd Consulting for Routine Care According to GOLD 2014 Classifications,” International Journal of Chronic Obstructive Pulmonary Disease 12 (2017): 1527–1537, 10.2147/COPD.S133793. PMC5447695 28579771

[19] G. A. Palmiotti , D. Lacedonia , V. Liotino , et al., “Adherence to GOLD Guidelines in Real‐Life COPD Management in the Puglia Region of Italy,” International Journal of Chronic Obstructive Pulmonary Disease 13 (2018): 2455–2462, 10.2147/COPD.S157779. PMC6101739 30147311

[20] N. Gupta , R. Haley , A. Gupta , and S. Sethi , “Chronic Obstructive Pulmonary Disease in the Intensive Care Unit: Antibiotic Treatment of Severe Chronic Obstructive Pulmonary Disease Exacerbations.” Seminars in Respiratory and Critical Care Medicine (Thieme Medical Publishers, 2020). 10.1055/s-0040-1708837 32781475

[21] K. A. Hasenstab , N. Yuan , T. Retson , et al., “Automated CT Staging of Chronic Obstructive Pulmonary Disease Severity for Predicting Disease Progression and Mortality With a Deep Learning Convolutional Neural Network,” Radiology: Cardiothoracic Imaging 3, no. 2 (2021): e200477, 10.1148/ryct.2021200477. PMC8098086 33969307

[22] S. Aissa , A. Knaz , J. Maatoug , et al., “Adherence of North‐African Pulmonologists to the 2017‐Global Initiative for Chronic Obstructive Lung Disease (GOLD) Pharmacological Treatment Guidelines (PTGs) of Stable Chronic Obstructive Pulmonary Disease (COPD),” BioMed Research International 2020 (2020): 1031845, 10.1155/2020/1031845. PMC7066397 32190644

[23] R. Ulmeanu , F. Mihaltan , O. Arghir , O. Mladinescu , and G. Teodorescu , “Treatment Goals in COPD: The Concordance Between Patients and Physicians (Interim Results of ACORD Study),” Chest 150, no. 4 (2016): 873A, 10.1016/j.chest.2016.08.973.

[24] R. Hough and A. Vora , “Crisis Management in the Treatment of Childhood Acute Lymphoblastic Leukemia: Putting Right What Can Go Wrong (Emergency Complications of Disease and Treatment),” Hematology 2017, no. 1 (2017): 251–258, 10.1182/asheducation-2017.1.251. PMC6142611 29222263

[25] P. A. Turan , O. Turan , F. Güldaval , C. Anar , G. Polat , and M. Büyükşirin , “Transitions Between COPD Groups: A Cross‐Sectional Study in Turkey,” Respiratory Medicine 178 (2021): 106310, 10.1016/j.rmed.2021.106310. 33529994

[26] K. K. Chan , F. Ko , H. S. Chan , et al., “Adherence to a COPD Treatment Guideline Among Patients in Hong Kong,” International Journal of Chronic Obstructive Pulmonary Disease 12 (2017): 3371–3379, 10.2147/COPD.S147070. PMC5713700 29238182

[27] S. P. Hogea , E. Tudorache , A. P. Fildan , O. Fira‐Mladinescu , M. Marc , and C. Oancea , “Risk Factors of Chronic Obstructive Pulmonary Disease Exacerbations,” Clinical Respiratory Journal 14, no. 3 (2020): 183–197, 10.1111/crj.13129. 31814260

[28] V. M. Sandu , T. Mihaescu , D. Filipeanu , A. Cernomaz , and R. A. Crisan‐Dabija , “Impact of Halotherapy on COPD Exacerbations,” European Respiratory Journal 54 (2019): PA2498.

[29] C. Asche , S. Leader , C. Plauschinat , et al., “Adherence to Current Guidelines for Chronic Obstructive Pulmonary Disease (COPD) Among Patients Treated With Combination of Long‐Acting Bronchodilators or Inhaled Corticosteroids,” International Journal of Chronic Obstructive Pulmonary Disease 7 (2012): 201–209, 10.2147/COPD.S25805. PMC3324995 22500120

[30] C.‐H. Chiang , S.‐L. Liu , C.‐H. Chuang , and Y.‐H. Jheng , “Effects of Guideline‐Oriented Pharmacotherapy in Patients With Newly Diagnosed COPD: A Prospective Study,” Wiener Klinische Wochenschrift 125 (2013): 353–361, 10.1007/s00508-013-0362-y. 23817861

[31] M. Miravitlles , A. Sicras , C. Crespo , et al., “Costs of Chronic Obstructive Pulmonary Disease in Relation to Compliance With Guidelines: A Study in the Primary Care Setting,” Therapeutic Advances in Respiratory Disease 7, no. 3 (2013): 139–150, 10.1177/1753465813484080. 23653458

[32] A. Duarte‐de‐Araújo , P. Teixeira , V. Hespanhol , and J. Correia‐de‐Sousa , “COPD: Analysing Factors Associated With a Successful Treatment,” Pulmonology 26, no. 2 (2020): 66–72, 10.1016/j.pulmoe.2019.05.012. 31221593

[33] N. Gupta , N. Malhotra , and P. Ish , “GOLD 2021 Guideliness for COPD—What's New and Why,” Advances in Respiratory Medicine 89, no. 3 (2021): 344–346, 10.5603/ARM.a2021.0015. 33881161

[34] S. Surani , A. Aiyer , S. Eikermann , et al., “Adoption and Adherence to Chronic Obstructive Pulmonary Disease GOLD Guidelines in a Primary Care Setting,” SAGE Open Medicine 7 (2019): 2050312119842221, 10.1177/2050312119842221. PMC6452427 31057794

[35] M. A. Khan , M. A. Khan , J. D. Walley , et al., “Feasibility of Delivering Integrated COPD‐Asthma Care at Primary and Secondary Level Public Healthcare Facilities in Pakistan: A Process Evaluation,” BJGP Open 3, no. 1 (2019): 18X101632. 10.3399/bjgpopen18X101632 PMC6480853 31049412

[36] V. Mangold , M. Boesing , C. Berset , et al., “Adherence to the GOLD Guidelines in Primary Care: Data From the Swiss COPD Cohort,” Journal of Clinical Medicine 12, no. 20 (2023): 6636, 10.3390/jcm12206636. PMC10607923 37892775

[37] M. M. Hassan , S. M. Sikandar , F. Jamal , M. Ameeq , and A. Kargbo , “Chronic Obstructive Pulmonary Disease Patients With Community‐Acquired Pneumonia on Inhaled Corticosteroid Therapy: A Comprehensive Analysis of Risk Factors, Disease Burden, and Prevention Strategies,” Health Science Reports 8, no. 1 (2025): e70395, 10.1002/hsr2.70395. PMC11770223 39872908

[38] M. Muneeb Hassan , M. Ameeq , F. Jamal , M. H. Tahir , and J. T. Mendy , “Prevalence of COVID‐19 among Patients With Chronic Obstructive Pulmonary Disease and Tuberculosis,” Annals of Medicine 55, no. 1 (2023): 285–291, 10.1080/07853890.2022.2160491. PMC9815254 36594409

[39] S. R. Singh , C. K. Billington , I. Sayers , and I. P. Hall , “Clonally Expanded Human Airway Smooth Muscle Cells Exhibit Morphological and Functional Heterogeneity,” Respiratory Research 15, no. 1 (2014): 57, 10.1186/1465-9921-15-57. PMC4014754 24886333

[40] A. Anzueto , S. Rogers , B. Donato , et al., “Treatment Patterns in Patients With Newly Diagnosed COPD in the USA,” BMC Pulmonary Medicine 24, no. 1 (2024): 395, 10.1186/s12890-024-03194-4. PMC11330015 39153976

[41] B. P. Yawn , P. L. Enright , R. F. Lemanske , et al., “Spirometry Can Be Done in Family Physicians' Offices and Alters Clinical Decisions in Management of Asthma and COPD,” Chest 132, no. 4 (2007): 1162–1168, 10.1378/chest.06-2722. 17550939

[42] N. Roche , C. Pribil , E. Van Ganse , et al., “Real‐Life Use of Fluticasone Propionate/Salmeterol in Patients With Chronic Obstructive Pulmonary Disease: A French Observational Study,” BMC Pulmonary Medicine 14, no. 1 (2014): 56, 10.1186/1471-2466-14-56. PMC3997842 24694050

[43] D. D. Sin and S. F. P. Man , “Chronic Obstructive Pulmonary Disease as a Risk Factor for Cardiovascular Morbidity and Mortality,” Proceedings of the American Thoracic Society 2, no. 1 (2005): 8–11, 10.1513/pats.200404-032MS. 16113462

[44] M. I. MacDonald , E. Shafuddin , P. T. King , C. L. Chang , P. G. Bardin , and R. J. Hancox , “Cardiac Dysfunction During Exacerbations of Chronic Obstructive Pulmonary Disease,” The Lancet Respiratory Medicine 4, no. 2 (2016): 138–148, 10.1016/S2213-2600(15)00509-3. 26781000

[45] J. L. Lopez‐Campos , B. Alcázar Navarrete , J. Soriano , et al., “Determinants of Medical Prescriptions for COPD Care: An Analysis of the EPOCONSUL Clinical Audit,” International Journal of Chronic Obstructive Pulmonary Disease 13 (2018): 2279–2288, 10.2147/COPD.S160842. PMC6067777 30100718

[46] C. H. Martinez , A. A. Diaz , A. D. Parulekar , et al., “Age‐Related Differences in Health‐Related Quality of Life in COPD,” Chest 149, no. 4 (2016): 927–935, 10.1016/j.chest.2015.11.025. PMC4944781 26836895

[47] M. Divo , C. Cote , J. P. de Torres , et al., “Comorbidities and Risk of Mortality in Patients With Chronic Obstructive Pulmonary Disease,” American Journal of Respiratory and Critical Care Medicine 186, no. 2 (2012): 155–161, 10.1164/rccm.201201-0034OC. 22561964

[48] P. J. Barnes and B. R. Celli , “Systemic Manifestations and Comorbidities of COPD,” European Respiratory Journal 33, no. 5 (2009): 1165–1185, 10.1183/09031936.00128008. 19407051

[49] A. Agustí , B. R. Celli , G. J. Criner , et al., “Global Initiative for Chronic Obstructive Lung Disease 2023 Report: GOLD Executive Summary,” Journal of the Pan African Thoracic Society 4, no. 2 (2022): 58–80.

[50] M. Gosho , T. Ohigashi , K. Nagashima , Y. Ito , and K. Maruo , “Bias in Odds Ratios From Logistic Regression Methods With Sparse Data Sets,” Journal of Epidemiology 33, no. 6 (2023): 265–275, 10.2188/jea.JE20210089. PMC10165217 34565762

[51] I. S. Tzeng , “To Handle the Inflation of Odds Ratios in a Retrospective Study WiTh a Profile Penalized Log‐Likelihood Approach,” Journal of Clinical Laboratory Analysis 35, no. 7 (2021): e23849, 10.1002/jcla.23849. PMC8274998 34043251

